# Midpalatal suture maturation staging using cone beam computed tomography in patients aged between 9 to 21 years

**DOI:** 10.1038/s41598-022-08293-y

**Published:** 2022-03-12

**Authors:** Fabio Savoldi, Ki Kwan Wong, Andy W. K. Yeung, James K. H. Tsoi, Min Gu, Michael M. Bornstein

**Affiliations:** 1 Orthodontics, Division of Paediatric Dentistry and Orthodontics, Faculty of Dentistry, The University of Hong Kong, Prince Philip Dental Hospital, 34 Hospital Road, Sai Ying Pun, Hong Kong SAR, China; 2 Department of Dentistry and Oral Health, La Trobe University, 109 Arnold St., Bendigo, VIC 3550 Australia; 3 Oral and Maxillofacial Radiology, Division of Applied Oral Sciences and Community Dental Care, Faculty of Dentistry, The University of Hong Kong, Prince Philip Dental Hospital, 34 Hospital Road, Sai Ying Pun, Hong Kong SAR, China; 4 Dental Materials Science, Division of Applied Oral Sciences and Community Dental Care, Faculty of Dentistry, The University of Hong Kong, Prince Philip Dental Hospital, 34 Hospital Road, Sai Ying Pun, Hong Kong SAR, China; 5 Department of Oral Health and Medicine, University Center for Dental Medicine Basel UZB, University of Basel, Mattenstrasse 40, CH-4058 Basel, Switzerland

**Keywords:** Developmental biology, Anatomy

## Abstract

Midpalatal suture was analysed with cone beam computed tomography to identify its maturation with respect to age and maxillary-complex growth in 72 patients 9- to 21-year-old. Maxillary-complex was divided in premaxillary, maxillary, and palatine segment. Interdigitation and ossification of midpalatal suture, its density relative to hard tissues and soft tissues, and midpalatal suture density ratio were measured for each segment. Correlation of each parameter with age and maxillary-complex length was analysed, and classification trees were developed for staging parameters. Midpalatal suture maturation stages (MPSMS, from A to E) were applied to assess relationship with age and maxillary-complex length. Regarding age, ossification increased in maxillary segment of males (*r*_*S*_ = 0.39, *p* = 0.032), while suture density relative to soft tissues increased in maxillary (*r*_*S*_ = 0.37, *p* = 0.042) and palatine segments (*r*_*S*_ = 0.41, *p* = 0.027) of males and in palatine segment of females (*r*_*S*_ = 0.32, *p* = 0.041). In males, suture density relative to soft tissues discriminated two age-stages (*p* = 0.024), and the same parameter (*p* = 0.023) paired with ossification (*p* = 0.027) discriminated two length-stages. MPSMS identified length-differences between stage A and B in females (*p* = 0.001). Midpalatal-suture ossification and its density relative to soft tissues showed some relationship with age and maxillary-complex length, especially in males. However, challenging staging and limitations in the imaging method may limit clinical applications.

## Introduction

Skeletal maturation refers to changes including shape modifications^1^, ossification of elements^2^, and density variations^3^. Although longitudinal size measurements provide valid growth representations^1^, they have limited predictive value when applied to cross-sectional assessment of individuals^4^. Thus, maturational assessments based on stages have been developed^1^. Despite assessment of the overall skeletal maturational may be valid for facial growth as well^4^, maxillary growth differs from mandibular growth and may deserve a specific assessment^5^, with the midpalatal suture having been proposed as a suitable structure for such purpose^6^.

Differences in some midpalatal suture parameters have been reported from puberty to adulthood^7–9^, providing the rationale for a possible maturational assessment. A first parameter is the interdigitation i.e., the ratio between the suture length and the distance between its beginning and end^10^, exhibiting the change from a flat interface in infancy to an interdigitated structure in adolescence^7^. A second one is the ossification i.e., the ratio between the ossified suture length and the suture length^9,10^, expressing the “fusion” that the suture begins during adolescence and that proceeds towards adulthood^9^. A third one is the density ratio i.e., the ratio between the grey density of the suture and a reference area^11^, representing both the increase in bone mineral density that happens during skeletal maturation^3^ and the sutural ligament ossification^9^. This said, the maxillary-complex is composed of premaxilla, maxilla, and palatine bones^12^, and the above-mentioned parameters should be measured for each of its segments^9^.

Analysing the midpalatal suture with cone beam computed tomography (CBCT) overcomes the need of biopsy for histology^7^ and micro-CT^13^, and a CBCT assessment based on multiple midpalatal suture maturation stages has been proposed^6^. However, previous studies have been unsupportive of its validity as a predictor of rapid maxillary expansion outcomes^11,14^, they showed a reliability not suitable for routine clinical application^14,15^, and considered its scientific basis as ill-founded^14^.

Currently, no substantial maturational assessment of the midpalatal suture exists, and it is questionable whether any could be—and should be—developed, given the hazard related to radiation exposure in growing patients^16^. Although growth studies based on lateral cephalometry can rely on longitudinal databases^17^ developed when the “as low as diagnostically acceptable being indication-oriented and patient-specific” (ALADAIP) principle^16^ was not in-force, there is no such archive for CBCT. Thus, the use of cross-sectional data of patients with different age offers the best growth estimate when CBCT is used, similarly to modern cephalometric growth studies^18^ or histological growth studies^7^.

The objective of the present study was to analyse the correlation between the main CBCT parameters of the midpalatal suture with age or maxillary-complex growth, from puberty to adulthood. Furthermore, the feasibility of creating maturational stages based on such parameters was assessed.

## Results

**Figure 1:**
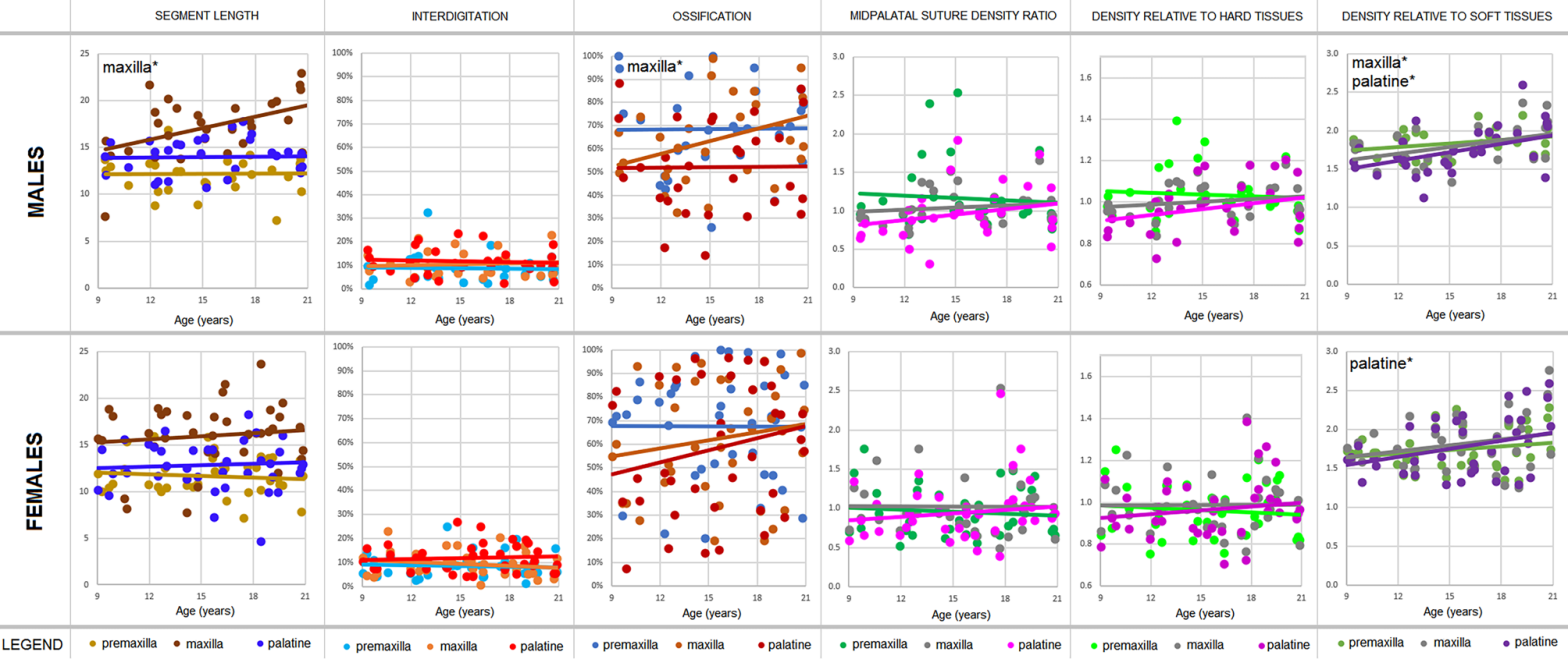
Correlations of each analysed midpalatal suture parameter with respect to age. Segment length is reported to show the regional growth. Data are presented for males (top) and females (bottom). Data of each suture segments (premaxilla, maxilla, palatine) are shown with different colours in each diagram (*for *p* < 0.05, **for *p* < 0.010, ***for *p* < 0.001).

**Figure 2:**
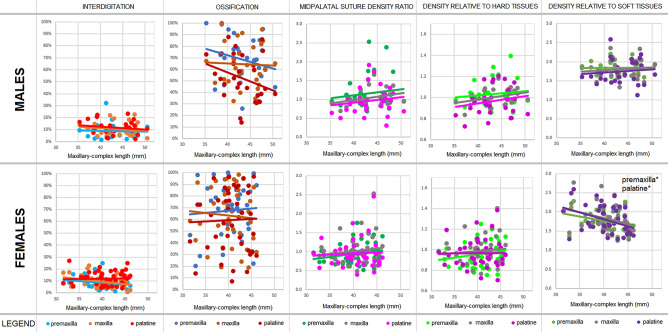
Correlations of each analysed midpalatal suture parameter with respect to maxillary-complex length, which represents the overall regional growth. Data are presented for males (top) and females (bottom). Data of each suture segments (premaxilla, maxilla, palatine) are shown with different colours in each diagram (*for *p* < 0.05, **for *p* < 0.010, ***for *p* < 0.001).

**Figure 3:**
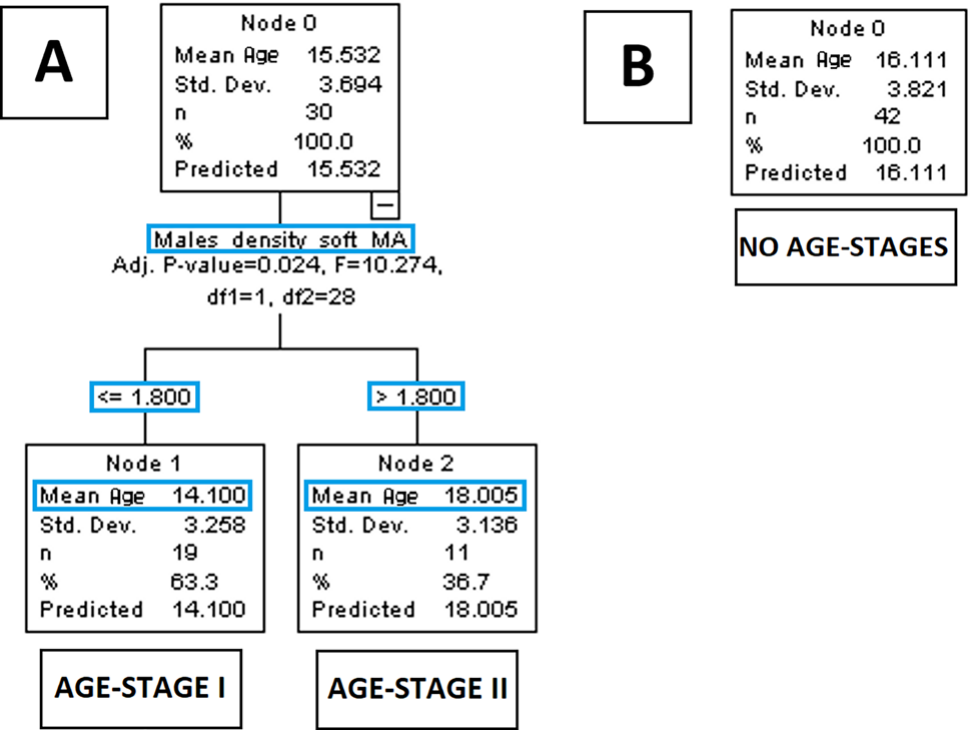
Staging of the midpalatal sure with respect to age by using automatic interaction detection. Classification trees based on age in males (**A**) and females (**B**) are displayed.

**Figure 4:**
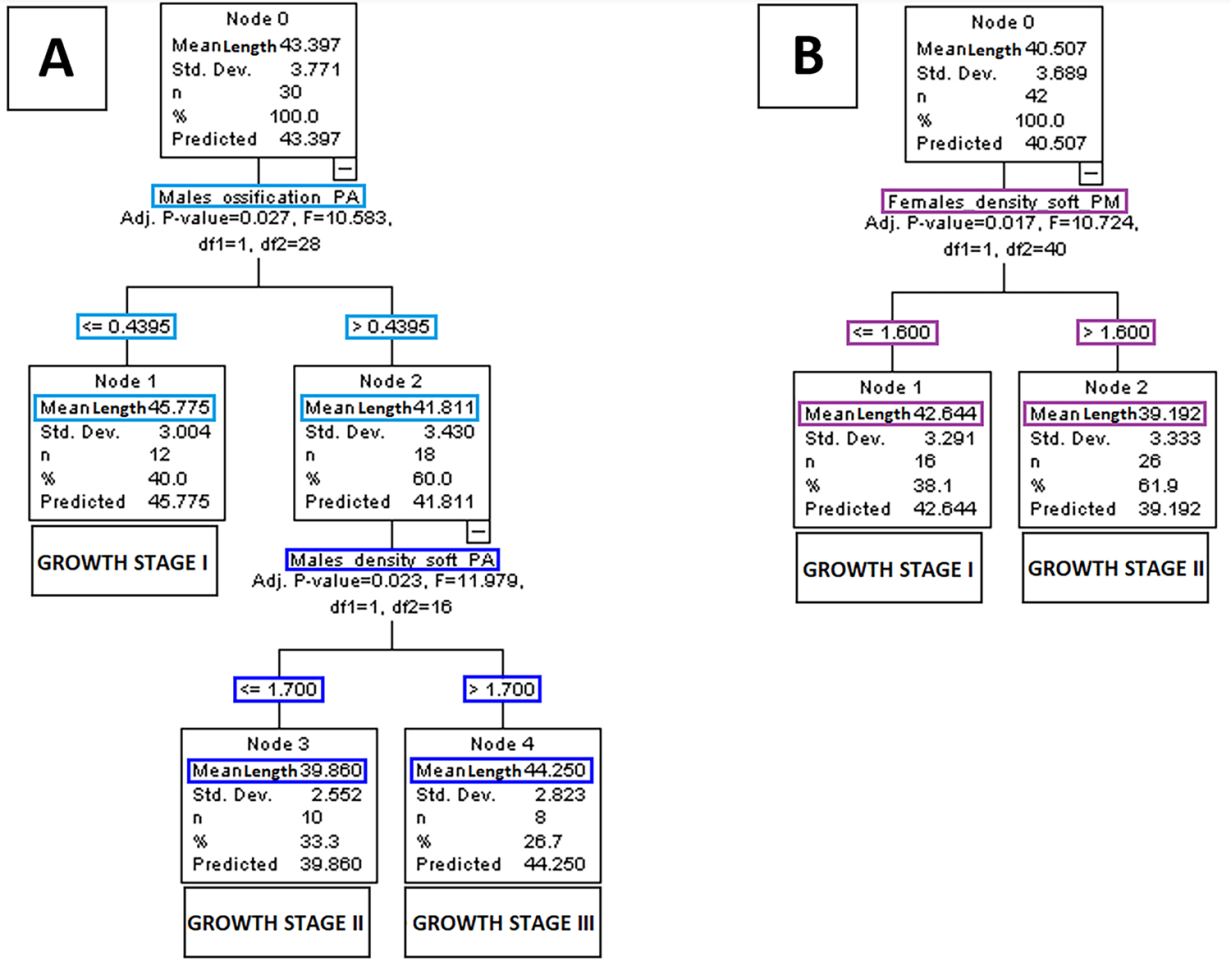
Staging of the midpalatal sure with respect to maxillary-complex growth by using automatic interaction detection. Classification trees based on maxillary-complex length (representing its growth) in males (**A**) and females (**B**) are displayed.

Correlations for each parameter are presented with respect to age (Fig. 1) and maxillary-complex length (Fig. 2). Staging of parameters with respect to age (Fig. 3) and maxillary-complex length (Fig. 4) is also presented.

### Method error and agreement

The intra-assessor agreement was excellent for all parameters (ICC from 0.915 to 0.997), beside ossification (ICC from 0.587 to 0.782) and interdigitation (ICC from 0.126 to 0.590). The method error was acceptable for segment length measurements (0.5 to 1.3 mm) and ratios (2 to 9%), beside ossification (13 to 14%). The intra-assessor agreement for the MPSMS was substantial (*k* = 0.649).

### Maxillary-complex length

The length of the maxillary-complex showed no variation with respect to age in females, meaning that no substantial growth was present. However, it showed a significant correlation in males (*r*_*S*_ = 0.39, *p* = 0.036), meaning that growth was more evident in this sex. In particular, the maxillary segment length showed a significant increase (*r*_*S*_ = 0.40, *p* = 0.030), meaning that this segment contributed to most of the maxillary-complex overall growth in males.

### Ossification

With respect to correlations with age, the ossification of the maxillary segment of males showed an increase (*r*_*S*_ = 0.39, *p* = 0.032), but not in females. However, no significant age-staging was possible.

With respect to correlations with maxillary-complex length, findings were not significant. Nevertheless, in terms of growth-staging, males showed two stages based on a cut-off value of 44% in the ossification of the palatine segment (*p* = 0.027).

### Suture density relative to soft tissues

With respect to correlation with age, the data of the density relative to soft tissues were similar in both sexes, showing an increase in all segments, which was significant in the palatine segment of females (*r*_*S*_ = 0.32, *p* = 0.041), and in the maxillary (*r*_*S*_ = 0.37, *p* = 0.042) and palatine segment (*r*_*S*_ = 0.41, *p* = 0.027) of males. In terms of age-staging, males showed two stages based on a cut-off value of 1.8 in the density relative to soft tissues of the maxillary segment (*p* = 0.024).

With respect to correlation with maxillary-complex length, no variation was present in males. Nevertheless, in terms of growth-staging, males showed two stages based on a cut-off value of 1.7 in the density relative to soft tissues of the palatine segment (*p* = 0.023). Although females showed some proportionality with respect to maxillary-complex length, no significant growth was present in this sex, and such statistically significant findings may have little clinical relevance.

### Interdigitation, midpalatal suture density ratio (MPSDR), and density relative to hard tissues

Interdigitation, MPSDR, and density relative to hard tissues findings were not significant for age or maxillary-complex length, and no significant staging was possible. Density relative to hard tissues data showed almost overlapping findings with MPSDR. In fact, this last parameter is the ratio between the suture grey density and the hard tissues grey density, to which the soft tissues grey density is subtracted (leading to a widening in the scattering of the values without affecting their trend).

### Midpalatal suture maturation stages (MPSMS)

With respect to age, the MPSMS did not show any significant difference among stages. With respect to growth, an increase of maxillary-complex length was present between stage A and B (*p* = 0.001), which was limited to females. None of the other CBCT parameters showed differences among stages (Table 1).

**Table 1:** Average values of age, maxillary-complex length, and CBCT parameters (interdigitation, ossification, midpalatal suture density ratio, midpalatal suture density relative to soft tissues, and midpalatal suture density relative to hard tissues) are presented for each of the five midpalatal suture maturation stages proposed by Angelieri et al*.* Values are reported independently for each segment of the midpalatal suture (premaxilla, maxilla, and palatine), and also for its total length. Furthermore, data are presented for the whole sample, limited to females, and limited to males.

Age (years)		Length (mm)				Interdigitation (%)				Ossification (%)				MPSDR				Density soft				Density hard			
		PM	MA	PA	Total	PM	MA	PA	Total	PM	MA	PA	Total	PM	MA	PA	Total	PM	MA	PA	Total	PM	MA	PA	Total
**All (n = 72)**																									
A	15.7	11.9	16.2	12.3	40.3	9	8	10	9	64	62	54	60	0.91	0.97	0.88	0.92	1.87	1.96	1.87	1.90	0.94	0.97	0.93	0.95
B	15.0	12.0	17.2	13.5	42.7	8	11	12	10	68	68	60	65	1.19	1.13	1.06	1.13	1.76	1.75	1.71	1.74	1.03	1.02	1.00	1.02
C	17.1	11.7	16.2	14.0	41.8	7	11	12	10	69	57	52	59	1.03	1.02	0.86	0.97	1.78	1.80	1.70	1.76	0.99	1.00	0.94	0.98
D	14.9	11.9	17.6	13.6	43.2	11	8	12	10	73	61	53	62	0.99	0.99	0.98	0.98	1.74	1.74	1.76	1.74	0.99	0.99	0.99	0.99
E	17.4	11.6	14.6	13.0	39.3	9	10	13	11	69	67	59	65	0.85	0.88	0.95	0.89	1.84	1.90	1.95	1.90	0.93	0.95	0.98	0.95
**Females (n = 40)**																									
A	15.2	11.8	14.8	10.6	**37.2**^**a**^	12	9	9	10	64	58	58	60	0.79	0.88	0.82	0.83	1.85	1.98	1.92	1.92	0.89	0.94	0.91	0.92
B	14.8	11.9	17.2	13.3	**42.4**^**a**^	7	10	12	9	70	71	72	71	1.03	1.22	1.20	1.15	1.69	1.80	1.78	1.75	0.98	1.05	1.04	1.02
C	17.8	11.2	16.0	14.3	41.4	6	10	12	9	68	57	54	60	1.05	1.04	0.87	0.98	1.76	1.79	1.70	1.75	0.99	1.00	0.94	0.98
D	13.6	12.3	17.8	13.2	43.3	9	5	14	9	71	58	44	58	0.98	0.98	0.82	0.93	1.62	1.62	1.54	1.59	0.98	0.98	0.93	0.96
E	18.1	10.9	14.8	12.6	38.3	10	10	15	12	67	70	58	65	0.84	0.87	0.98	0.90	1.82	1.88	1.96	1.88	0.93	0.95	0.99	0.96
**Males (n = 32)**																									
A	16.6	11.9	18.6	15.4	45.9	6	6	11	7	66	68	47	60	1.13	1.14	0.98	1.09	1.89	1.92	1.77	1.86	1.02	1.03	0.96	1.00
B	15.2	12.0	17.3	13.7	42.9	9	12	13	11	66	66	52	61	1.31	1.07	0.96	1.11	1.81	1.72	1.65	1.73	1.07	1.01	0.97	1.02
C	15.1	13.0	16.8	13.1	42.9	9	13	11	11	72	57	45	58	0.99	0.97	0.84	0.93	1.85	1.83	1.71	1.80	0.99	0.98	0.92	0.97
D	16.2	11.6	17.5	14.1	43.1	12	10	11	11	75	63	62	67	1.00	0.99	1.13	1.04	1.85	1.86	1.97	1.90	1.00	1.00	1.06	1.02
E	15.2	13.7	14.2	14.2	42.1	5	10	6	7	77	58	64	66	0.86	0.91	0.86	0.88	1.92	1.98	1.92	1.94	0.93	0.96	0.93	0.94

*PM* premaxillary segment, *MA* maxillary segment, *PA* palatine segment, *MPSDR* midpalatal suture density ratio, *Density soft* density of the midpalatal suture relative to the soft tissues, *Density hard* density of the midpalatal suture relative to the hard tissues.

Stages were compared with Kruskal–Wallis one-way ANOVA with Mann–Whitney U post hoc test (adjusted significance α = 0.005).

Statistically significant differences between stages are reported with the same superscript letter.

## Discussion

Despite growth being a 3D process, length increase on one characteristic direction has been used for representing skeletal growth^19,20^. The maxillary-complex growth progresses quite similarly in both antero-posterior and latero-lateral directions until about 15 years in females and 17 years in males^19,20^. Thus, changes in the antero-posterior length offer a reasonable estimation of its overall growth^5,19^. In fact, sexual dimorphism was found in the present study, supporting its clinical validity^5,19^, as the antero-posterior growth of the maxillary-complex mostly happens through the posterior transverse suture, which is active until the age of 13 in females and 15 in males^7^. The present findings showed that maxillary-complex growth was mainly related to the maxilla, while the length of premaxilla and palatine bones was quite stable in both sexes after the age of 9.

Ossification of the midpalatal suture can be measured on a plane either parallel to the hard palate or perpendicular to it^9,10^. In the present work, it was measured on a parallel plane for coherence with previous studies^6,11,21^. Despite ossification increased with age in the maxillary segment of males, marked ossification is uncommon during adolescence and it may substantially increase only after 20-year-old^9^. Furthermore, a great variability of this parameter was reported with respect to age^8,9^. No significant trend and relevant variability were also observed between ossification and maxillary-complex length, making its relationship with growth questionable. However, the combination of ossification with other factors such as bone density may become relevant^8^. In fact, the ossification of the palatine segment contributed to predict maxillary-complex length in males when associated with the density of the suture relative to soft tissues.

After the age of 9, both sexes showed increased suture density relative to soft tissues with respect to age, which was particularly evident in the palatine segment. Accordingly, the bone mineral density gradually increases from infancy to late adolescence, and tends to stabilise for a certain period during early adulthood^3^. When the same parameter was related to the maxillary-complex length, relatively stable values were present in males, while some negative correlation emerged in females. Even though such density decrease may be due to earlier sutural remodelling in the sex reaching skeletal maturation first^2^, almost no growth was present in females and these findings may have limited meaning. Furthermore, staging of this parameter would be necessary for clinical applications, but the density of the sutural area relative to soft tissues is not suitable as hand-wrist^2^ or vertebrae^1^ for such staging process. In fact, the grey-density is a continuous variable that does not allow an easy discretisation based on shape variation or other qualitative changes. In addition, although the use of a ratio—compared to a simple grey density—may reduce the limitations of CBCT in measuring bone mineral density^11^, the validity of grey density values with respect to Hounsfield units is still limited^22^. This said, a cut-off value of 1.8 in the maxillary segment of males allowed to discriminate between younger and older patients, and a cut-off value of 1.7 in the palatine segment allowed to differentiate between a shorter and a longer maxillary-complex. Thus, despite the use of the suture density relative to soft tissues per se might be limited, it may be one of the parameters constituting a multi-modal maturational assessment^21^, as it represents the regional bone mineral density, which is relevant to skeletal growth^3^.

With regard to the previously proposed MPSMS method, age did not show any difference among its stages, and difference in maxillary-complex length were present only between stage A and B (limited to females). Since no CBCT parameter showed any difference among the proposed five stages, the scientific rationale of this method is questionable. Given the unsupportive reports of its clinical validity as outcome-predictor of midpalatal suture expansion^11,14^ and its limited reliability^14,15^, adoption of other well-established growth assessment methods is advisable in clinical practice^1,2^.

Despite investigations of the maxillary-complex growth ideally requires longitudinal assessments, cross-sectional studies are probably the best available evidence when CBCT imaging is used^7,18^, due to concerns of exposing young patients to ionising radiations multiple times during growth^16^. It is unclear whether this limitation led to the lack of a significant correlation between age and maxillary length in females, despite maxillary size should increase up to 15 years^19,20^.

In addition, understanding the validity of the analysed parameters as predictors of maxillary expansion^11^ was beyond the scope of the present study, which focused on the feasibility of staging such parameters—a prerequisite that is needed before any validity assessment.

Further studies with larger FOV including the neck may allow to compare the analysed parameters with skeletal growth assessed via cervical vertebral maturation^1^, despite the ALADAIP principle limits the use of such large FOV in children^16^.

Additional investigations using CBCT with large FOV may also include in the analysis the effect of facial skeletal morphology on midpalatal suture maturation^23^, which may act as a confounding factor.

In conclusion, limited to CBCT imaging, and to the 9 to 21-year-old age-range:

- Interdigitation, midpalatal suture density ratio, and density of the sutural area relative to hard tissues may not be useful indicators of midpalatal suture maturation.
- Ossification was correlated with age in males, and density of the sutural area relative to soft tissues was correlated with age in both sexes.
- In males, the density of the sutural area relative to soft tissues may identify two age-stages, while paired with ossification it may identify three growth-stages of the maxillary-complex.
- Given the intrinsic methodological limitations of CBCT imaging and the difficult staging of the proposed variables, caution is advisable before applications of CBCT-based assessment methods of midpalatal suture maturation to clinical settings.

## Methods

### Sample selection

Patients with CBCT performed for diagnosis of tooth impaction, cysts, and dental abscesses were screened. CBCTs were acquired with two machines (Planmeca ProMax 3D^©^, Planmeca, Finland, and i-CAT^©^, KaVo Dental, US), and those with similar voltage (90–120 kV) and resolution (200–400 μm), and with the FOV (from 8 × 5 to 20 × 18 cm) including both hard palate and lateral pterygoid muscle were selected. Patients with cleft palate, craniofacial syndromes, pathologies affecting the sutural area, orthognathic surgery, orthodontic treatment, or showing image artefacts were excluded. The sample size calculation was based on the ability to detect a moderate bivariate correlation (*r*_*S*_ ≥ 0.5)^24^ with power *β* = 0.8 and significance *α* = 0.05, requiring a minimum of 26 patients. Random sampling stratified by age was used to select patients from the CBCT archive and achieve a uniform age distribution, resulting in a sample of 72 patients (30 males and 42 females) (Table 2). All methods were carried out in accordance with relevant guidelines and regulations. Approval was obtained by the Institutional Review Board of the University of Hong Kong/Hospital Authority Hong Kong West Cluster (UW-19-219) that allowed informed consent to be waived due to the retrospective nature of the study and because the analysis used anonymous clinical data. The protocol was registered in the Clinical Trial Registry of the University of Hong Kong (HKUCTR-2883).

**Table 2:** Sample characteristics.

Age range (years)	All	Females	Males
	n	n	n
9 to 11	12	7	5
12 to 13	12	5	7
14 to 15	12	8	4
16 to 17	12	6	6
18 to 19	12	9	3
20 to 21	12	7	5
Total	72	42	30

### Image acquisition and analysis

**Figure 5:**
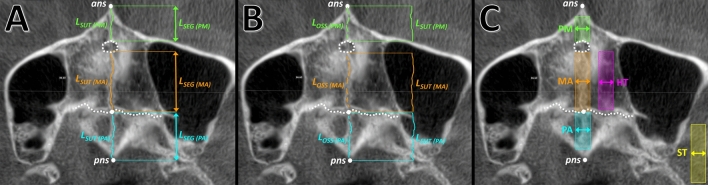
Description of the anatomical assessment of the midpalatal suture on CBCT images. Anterior nasal spine (*ans*), posterior nasal spine (*pns*), incisal canal (dashed circle), and posterior transverse suture (dashed line) are indicated in white. Suture length (*L*_*SUT*_) and segment length (*L*_*SEG*_) (**A**). Suture length (*L*_*SUT*_) and ossified suture parts (*L*_*OSS*_) (**B**). Areas in which the grey density was measured for representing the hard tissues (HT), and the soft tissues (ST), arrows indicate a 4.0 mm width (**C**). Measurements are shown in different colours for each suture segment: premaxilla (PM), maxilla (MA), and palatine (PA).

CBCTs were oriented with a computer software and the slice thickness was standardised at 400 μm for uniform assessment (Planmeca Romexis^©^ 5.0, Planmeca, Finland). Anterior nasal spine (*ans*, defined as the most anterior point of the premaxilla on the midsagittal plane) and posterior nasal spine (*pns*, defined as the most posterior point of the palatine bones on the midsagittal plane) were identified. Each volume was oriented such that the coronal slice was aligned to the posterior transverse suture, the sagittal slice was aligned to *ans-pns*, and the axial slice was aligned with the hard palate and with *ans-pns*. The slice centred in the hard palate thickness was identified (Fig. 5) and length (mm), area (mm^2^), and density (grey scale from 0 to 256 bit) measurements were analysed with an additional computer software (ImageJ). This software allowed to manually select an area of interest of the desired size and automatically calculated the average grey density, while the CBCT software did not have such function. Measurements were taken by a primary assessor (K.K.W.) after calibration with a secondary assessor (F.S.) on five CBCTs. After a wash out period of about one month, measurements were repeated on thirty patients. Images were coded and assessors were blinded to age and sex of patients.

### Variables

The total maxillary-complex length (from *ans* to *pns*), which was used as estimate of its growth^5^, and the length of premaxillary (from *ans* to incisal canal), maxillary (from incisal canal to posterior transverse suture), and palatine (from posterior transverse suture to *pns*) segments was measured (Fig. 5). The interdigitation was calculated as the ratio between the actual suture length and segment length, and the ossification as the ratio between the sum of ossified suture parts and suture length. The average grey density of the suture (*GD*_*SUT*_) was measured in an area with length equal to the investigated segment and 4.0 mm wide. A second area with length equal to the maxillary segment and 4.0 mm wide was measured in the hard palate, representing hard tissues (*GD*_*HARD*_). A third area of the same size was measured in the lateral pterygoid muscle, representing soft tissues (*GD*_*SOFT*_) (Fig. 5). Compared to the former study, the width of the sampling area for the suture was reduced from 6.0 to 4.0 mm (making it more representative of the suture and reducing the influence of surrounding tissues), and the sampling area for the soft tissues was taken from the external pterygoid muscles instead of the soft palate (as the soft palate may be not clearly visible on axial CBCT slices). The midpalatal suture density ratio (MPSDR) was calculated as (*GD*_*SUT*_–*GD*_*SOFT*_)/(*GD*_*HARD*_–*GD*_*SOFT*_)^11^. The suture grey density relative to hard tissues (*SGD*_*HARD*_) was calculated as *GD*_*SUT*_/*GD*_*HARD*_, and relative to soft tissues (*SGD*_*SOFT*_) as *GD*_*SUT*_/*GD*_*SOFT*_. The midpalatal suture maturation stages (MPSMS) were measured^6^. The stages should represent progressive maturation of the midpalatal suture in terms of shape, grey density, interdigitation, and ossification in different areas, varying from A (straight high-density line with no or little interdigitation), B (scalloped high-density line), C (two parallel, scalloped, high-density lines close to each other and separated in some areas by small low-density spaces), D (fusion completed in the palatine bone), and E (fusion anteriorly in the maxilla).

### Statistical analysis

For each parameter, Spearman’s coefficient (*r*_*S*_) was used to assess correlations with age and maxillary-complex length. For each sex, two classification trees were developed (one based on age, and one based on maxillary-complex length) using Chi-square automatic interaction detection (CHAID) for staging the parameters. Each parameter was compared among the five MPSMS by using Kruskal–Wallis one-way ANOVA, and Mann–Whitney U post hoc test (adjusted significance α = 0.05/10 = 0.005).

Intraclass correlation coefficient (ICC) was used to calculate the intra-assessor agreement. Cohen’s *k*-coefficient was used to calculate the intra-assessor agreement of the MPSMS. The method error was estimated with Dahlberg’s formula. Data were analysed with a statistical software (SPSS^©^ V23.0, IBM, USA) with significance α = 0.05.

## Supplementary Information

Supplementary Information.

## Data Availability

All data generated or analysed during this study are included in this published article and its Supplementary Information file.
